# Breastfeeding after return to work: An Australian national workplace survey

**DOI:** 10.1111/mcn.13516

**Published:** 2023-04-04

**Authors:** Elaine Burns, Emma Elcombe, Heather Pierce, Sky Hugman, Susanne Gannon

**Affiliations:** ^1^ School of Nursing and Midwifery Western Sydney University Sydney New South Wales Australia; ^2^ Ingham Institute for Applied Medical Research Western Sydney University Sydney New South Wales Australia; ^3^ School of Social Sciences Western Sydney University Sydney New South Wales Australia; ^4^ School of Education Western Sydney University Sydney New South Wales Australia

**Keywords:** breastfeeding, employment, gender equity, human rights, survey, workplace

## Abstract

Breastfeeding initiation rates in Australia are high but duration rates fall well below the World Health Organization targets. Return to work is a known factor impacting 6 months exclusive breastfeeding and continuation into the infants second year of life. Work related factors can influence a woman's confidence in maintaining breastmilk supply after return to employment and determine whether she meets her personal breastfeeding goals. This cross‐sectional online survey is the first Australian study to explore women's experience of maintaining breastfeeding after return to work, in all work sectors. Results revealed variations across work sectors reflected in worker autonomy and confidence in speaking up about breastfeeding rights. Women who had autonomy or flexibility in planning their workday were more likely to be confident in maintaining breastmilk supply. The main predictors for milk supply confidence and meeting personal breastfeeding goals included having: a suitable place to express milk; confidence in speaking out about rights; a formal return‐to‐work plan; a supportive workplace; and returning to work after the period of exclusive breastfeeding. This study reveals that supportive workplace environments can lead to increased confidence in maintaining milk supply, extending durations of breastfeeding. Women who are confident in their rights to express breastmilk, or breastfeed at work, are more likely to meet their own breastfeeding goals. Education, and awareness raising, on the rights of breastfeeding women in the workplace, is a gender equity imperative that can improve experiences for breastfeeding women, and, increase manager and co‐worker knowledge for creating enabling workplace environments for breastfeeding employees.

## Key messages

- Return to work after the birth of a baby impacts breastfeeding duration and personal goal attainment. Unsupportive employment factors can lead to a loss of confidence in ability to maintain adequate breastmilk supply.
- Having access to sufficient paid parental leave, a suitable place to express milk, autonomy, or ability to take lactation breaks, a formal return to work plan, and confidence in speaking up about breastfeeding rights, impacts meeting breastfeeding goals.
- Increasing knowledge and awareness on the rights of breastfeeding workers, and the components of supportive and enabling environments, will improve women's confidence in their ability to maintain adequate breastmilk supply, and meet their breastfeeding goals, after return to work.

## 1 INTRODUCTION

In Australia, new mothers' desire to breastfeed is reflected by initiation rates of 93%–96%. Yet the duration of exclusive breastfeeding to 5 months is low at 15%–22% and breastfeeding to 2 years of age is 7%–10% (AIHW, 2011; Netting et al., 2022; Scott et al., 2019). Australian breastfeeding rates are well below the World Health Organization's (WHO) global nutrition targets of 50% exclusive breastfeeding rates by 2025 (WHO/UNICEF, 2014). One key factor impacting exclusive breastfeeding rates, to 6 months and beyond, is the breastfeeding mother's Return To Work (RTW) (Bai & Wunderlich, 2013; Scott et al., 2019; Smith, McIntyre, et al., 2013). Australian research by Xiang and colleagues (2016) revealed that women who RTW within 3 months of having their baby had lower probability of breastfeeding at 6 months, compared to those who had not returned to work. The likelihood of predominantly breastfeeding has also been shown to decline if there is RTW within the first 6 months after birth (Xiang et al., 2016). Even at the beginning of a breastfeeding journey, return to work plans have been shown to impact planned duration of breastfeeding, due to decreased confidence in maintaining milk supply after RTW (Smith, Javanparast, et al., 2017; Thomas‐Jackson et al., 2016). There is a known association between RTW and breastfeeding cessation, especially when RTW is before 6 months and/or when the woman works full‐time (Cooklin et al., 2012; Smith, Javanparast, et al., 2017; Smith, McIntyre, et al., 2013; Weber et al., 2011; Xiang et al., 2016). In Australia, a national paid parental leave scheme is available for eligible women to access up to 18 weeks of paid maternity leave (Services Australia, 2022). Whilst Australian workplaces appear to promote family friendly policies, women experience variable levels of support for breastfeeding as reported by university staff, hospital employees, and police officers (Burns & Triandafilidis, 2019; Gilmour et al., 2013; Newton & Huppatz, 2020; Smith, Javanparast, et al., 2017; Smith, McIntyre, et al., 2013; Weber et al., 2011). This study is the first in Australia to explore the experiences of women across all 19 Australian Bureau of Statistics workplace classifications (ABS, 2006).

Support for breastfeeding employees is essential to improve breastfeeding outcomes. International systematic review of workplace support for breastfeeding women, included 22 research papers from the United States, Taiwan, Indonesia, Malaysia, Thailand, Iran, Ghana, United Kingdom, Brazil and Puerto Rico. The review revealed the most common supports available for breastfeeding women were the provision of a space to express or pump milk, and/or lactation breaks. Access to a designated space led to longer durations of ongoing breastfeeding (Dinour & Szaro, 2017). Without access to space, and other provisions for breastfeeding needs, many women will cease breastfeeding before they had planned (Vilar‐Compte et al., 2021; Wallenborn et al., 2019).

Unmet breastfeeding goals can lead to feelings of guilt and anxiety and adversely impact the mental health of new mothers (Brown, 2018; Dixit et al., 2015; Fahlquist, 2016). Dixit and colleagues (2015) report that health professionals who RTW and were unable to achieve their personal breastfeeding goals felt “…sad, devastated, defeated, angry, like a failure, and inadequate…” (p. 244). Maintaining adequate breastmilk supply was a key concern after RTW (Dixit et al., 2015). The risk of postnatal depression increases when personal breastfeeding goals cannot be met (Borra et al., 2015; Gregory et al., 2015). Regardless of the breastfeeding duration goal set by women, attainment of the goal generates feelings of pride, improved confidence and a sense of achievement, especially if there were multiple challenges to overcome (Brown & Lee, 2011; Shepherd et al., 2017).

Greater understanding of workplace factors that increase a woman's confidence in maintaining breastmilk supply, and reaching personal breastfeeding goals, is needed. There is currently a lack of contemporary Australian research on RTW experiences of breastfeeding employees across a variety of work sectors.

### 1.1 Aims and objectives

This study aimed to explore the workplace provisions for Australian women who RTW and maintain breastfeeding after the birth of a baby.

The objectives of this study were to:

1. Explore the workplace‐related factors that impacted confidence in maintaining breastmilk supply after RTW;
2. Explore workplace‐related facilitators and barriers to achieving personal breastfeeding goals; and
3. Examine the difference in confidence in breastmilk supply, and breastfeeding goal attainment, across all work sectors.

## 2 METHOD

### 2.1 Study design

This cross‐sectional study used an online survey to explore the impact of RTW on breastfeeding confidence, maintaining breastmilk supply, and achieving breastfeeding goals, across all Australian work sectors. The study received ethics approval from Western Sydney University (H13067) and support for advertising from the Australian Breastfeeding Association (ABA Approval No. 2019‐10).

### 2.2 Data collection

Qualtrics online survey software was used to generate the survey, which was distributed during World Breastfeeding Week, 1st–31st August 2019. Snowball sampling was used and the survey was distributed freely through social media networks (Facebook, Twitter) and via the Australian Breastfeeding Association ‘member‐only’ online and facebook groups. The inclusion criteria for the study was: women who had RTW while breastfeeding in Australia within the last 3 years.

### 2.3 Survey instrument

Survey questions included demographics such as age, country of birth, highest level of education, living in an urban or rural environment and whether breastfeeding was supported at home. Work sector and employment characteristics, and details of participants' recent experiences of breastfeeding and RTW were also collected. Questions were adapted from an existing validated US workplace breastfeeding support scale incorporating questions on common factors such as written policy, designated space, lactation room features (Bai, Peng, et al., 2008). Additional items were added to the survey based on the work of US researchers Greene and Olson (2008) such as: work culture, manager support, co‐worker support and physical environment. Wording of the items were modified for Australian conventions and context. Survey questions were also informed by the themes that emerged from our earlier qualitative analysis of Australian women's experiences of RTW and breastfeeding (RTW&BF) (Burns et al., 2022). This informed the importance of questions on ‘confidence with maintaining milk supply’ after RTW and ‘breastfeeding goal’ attainment. Questions were pilot tested with 12 breastfeeding women, colleagues and ABA volunteers. After pilot testing additional questions were added on workplace pride in supporting breastfeeding, and the perceived impact of breastfeeding on workplace promotion opportunities.

#### 2.3.1 Work characteristics

Workplace was identified in the survey according to industry, as classified by the Australian Bureau of Statistics (ABS) Australian and New Zealand Standard Industrial Classification (cat. no. 1292.0) (ABS, 2006). Due to the high number of ABS categories (*n* = 19) for work sectors, and to facilitate analysis, we sub‐grouped industry classifications according to gender (ABS, 2020). Two industries were dominated by female workers: ‘Healthcare and Social Assistance’ and ‘Education and Training’. Industries classified as having more females than males were grouped as one category called: ‘Administrative and support services’ (including ‘Retail trade’; ‘Accommodation and food services’; and ‘Rental, hiring and real estate services’). Those industries classified as having more males than females were also grouped as one category called: ‘Professional, scientific and technical services’ (including ‘Financial and insurance services’; ‘Arts and recreation services’; ‘Public administration and safety’ and ‘other services’). Finally, predominantly male work sectors were combined into one category: ‘Mining’ & ‘Manufacturing’ (including ‘Agriculture, Forestry and Fishing’; ‘Electricity, gas, water and waste services’; ‘Construction’; ‘Transport, postal and warehousing’; ‘Information, media and telecommunications’ and ‘Wholesale trade’). Co‐worker and manager gender were identified by responses to survey questions asking whether co‐workers or managers were ‘mostly male’, ‘mostly female, or ‘equally male and female’. Work status was identified as ‘Full‐time’, ‘Part‐time’, ‘Casual’, with missing data (*n* = 10) categorised as ‘not currently in employment’. Worker autonomy was identified by asking women to rate on a scale the following statement: “I have autonomy in planning my day”, from ‘all of the time’ to ‘none of the time’. The scale was converted to a binary outcome, ‘yes’ or ‘no’, with ‘unsure’, ‘some of the time’ and ‘none of the time’ categorised as ‘no’.

#### 2.3.2 Workplace provisions to support breastfeeding

For workplace ‘provision’, women were asked whether their workplace had a formal RTW plan, whether breastfeeding was included in the plan and whether the workplace provided childcare. For these questions the response options included ‘yes’, ‘no’ or ‘unsure’ (we categorised ‘unsure’ as ‘no’). For those who expressed breastmilk (EBM) at work, participants were asked whether they had a suitable space to undertake this activity. Participants could indicate: if a space was provided, if it was comfortable, and met their needs. Participants who reported that they had a place that met their needs were categorised as ‘having a suitable place to EBM’.

#### 2.3.3 Workplace culture

We explored workplace ‘culture’ by asking whether the workplace took pride in, or promoted, the importance of RTW&BF, and the woman's breastfeeding rights. For these questions, response options included ‘yes’, ‘no’ or ‘unsure’ (we categorised ‘unsure’ as ‘no’). We asked respondents their opinion on whether peers and managers had knowledge about women's rights to breastfeed, EBM, and take lactation breaks at work. Responses were converted into a binary outcome, ‘yes’ or ‘no’, where response options ‘unsure’, ‘no knowledge’ or ‘very limited knowledge’ were categorised as ‘no’. Women were also asked whether their managers and co‐workers were supportive of their decision to RTW and maintain breastfeeding, with response options offered as ‘yes’, ‘no’ or ‘others were not aware that I was breastfeeding/EBM’. Women's level of confidence in speaking about their rights when choosing to RTW and maintain breastfeeding was measured on a scale 0 to 100, where ‘0’ was ‘Reluctant’ and ‘100’ was ‘Very confident’.

### 2.4 Statistical analyses

Survey data were cleaned and sorted using Excel, and then entered into IBM SPSS Statistics (vs. 26). Means and standard deviations describe the continuous data while categorical data is described using frequencies and percentages. To assess differences between groups Chi Square was used for categorical data, and independent sample *t*‐tests or Mann–Whitney *U* (MW‐U) tests were used for continuous data. The key outcome variables of ‘Confident in maintaining milk supply on RTW’ and ‘Met breastfeeding goals’ were assessed in relation to demographic and workplace variables individually (Table 4). With the exception of ‘Age of child at BF cessation’, which was missing a significant amount of data, those variables shown to be associated with a key outcome (significance level <0.07) were then included in a multivariable logistic regression model (model a) to assess which of these variables were most strongly associated with the key outcome. Due to the relatively small number of responses for ‘Length of time EBM at work’ and ‘Child age when EBM was no longer needed’ a second pair of regressions were completed (model b), where these two variables were removed from the analysis for the purposes of increasing the power of the analyses and removing any potential confounding factors that these variables might have on relationships between workplace conditions and policies and the primary outcomes variables. The step‐backward logistic regressions were completed in R v4.1.2 using packages: stats and MASS. This analysis initially computed the full model using the glm function. The step.backward function (Venables & Ripley, 2002) then iteratively removes the variable contributing least to model fit. This iteration continues until only those variables significantly contributing to the model remain. The ‘step.backward’ package uses AIC to assess model fit. The reported models show the outcomes after variable removal.

## 3 RESULTS

**Figure 1:**
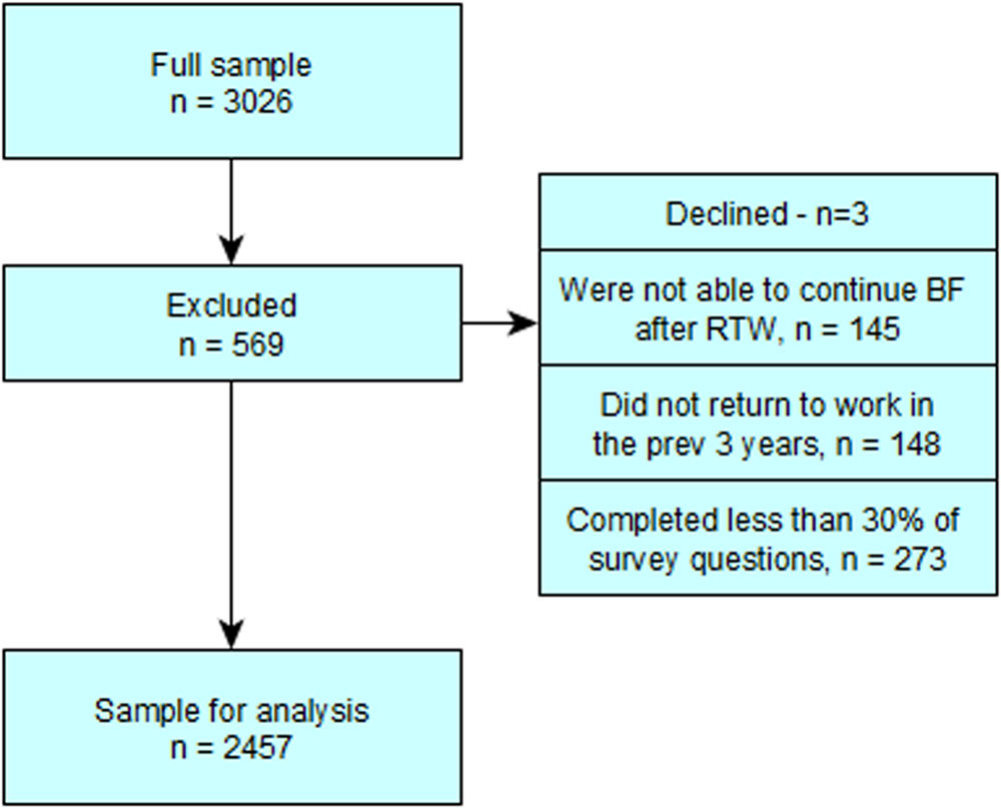
Flowchart of participants.  Those who did not complete at least 30% of the survey missed several key questions therefore their data was excluded.

A total of 3026 participants responded to the survey. Four‐hundred and twenty‐one participants were excluded because they: declined to participate (*n* = 3), did not meet inclusion criteria (*n* = 293), or did not complete at least 30% of the survey (*n* = 273) (see Figure 1).

### 3.1 Participant demographics

The mean age of participants was 33.0 years (*SD* = 4.34, range: 19–52 years). Most participants were born in Australia (86.5%), and more than one in three worked in the Healthcare & Social Assistance sector (35.6%). Most lived in a suburban environment (51.3%), while the rest lived in rural (26.8%) and urban (21.9%) areas. Participants in urban areas were more likely to be working in the Professional and Financial sector than in the Mining and Manufacturing sector (28.1% vs. 16.8%). Participants represented all Australian States and Territories, with the majority from New South Wales (28.6%), Victoria (22.0%) and Queensland (20.2%). Most participants (91.5%) had a post‐school qualification (certificate, diploma, or degree). Participants working in Administrative and Social Services and Mining and Manufacturing sectors were more likely to have high school level qualifications (27.9% and 15.1% vs. 9.7% for the full sample). See Table 1.

**Table 1:** Participant demographics.

	Healthcare & Social Assistance (*n* = 874)		Education & Training (*n* = 424)		Administrative & Support Services (*n* = 337)		Professional & Financial (*n* = 669)		Mining & Manufacturing (*n* = 152)		Full sample (*n* = 2457)	
Characteristic	*n*	%	*n*	%	*n*	%	*n*	%	*n*	%	*n*	%
**Country of birth (*n* = 2454)**												
Australia	769	88.0	371	87.7	302	89.6	555	83.1	125	82.2	2122	86.5
Other	105	12.0	52	12.3	35	10.4	113	16.9	27	17.8	332	13.5
**State or territory (*n* = 2315)**												
New South Wales	242	30.1	132	33.2	96	28.8	193	30.5	39	26.4	702	30.3
Victoria	213	26.5	87	21.9	63	18.9	141	22.3	37	25.0	541	23.4
Queensland	172	21.4	88	22.2	83	24.9	118	18.6	36	24.3	497	21.5
Western Australia	60	7.5	27	6.8	26	7.8	37	5.8	19	12.8	169	7.3
South Australia	57	7.1	24	6.0	26	7.8	47	7.4	10	6.8	164	7.1
Australian Capital Territory	15	1.9	14	3.5	12	3.6	65	10.3	1	0.7	107	4.6
Northern Territory	31	3.9	18	4.5	15	4.5	18	2.8	2	1.4	84	3.6
Tasmania	14	1.7	7	1.8	12	3.6	14	2.2	4	2.7	51	2.2
**Geographical remoteness (*n* = 2343)**												
Urban	192	23.4	68	17.0	50	14.9	179	28.1	25	16.8	514	21.9
Suburban	384	46.8	213	53.3	186	55.5	337	52.8	81	54.4	1201	51.3
Rural	245	29.8	119	29.8	99	29.6	122	19.1	43	28.9	628	26.8
**Highest education (*n* = 2454)**												
School qualification	20	2.3	5	1.2	95	28.2	65	9.7	23	15.1	208	8.5
Post‐school qualification	853	97.6	419	98.7	242	71.9	603	90.1	129	84.8	2246	91.5

*Note*: Participants were on average 32.97 years old (*SD* = 4.38), participant age differed by industry (*F* = 21.7, *df* = 4,2445, *p* < 0.001). Analysis of variance found participants working in the Administration sector were on average 1.5 years younger (*t* = −5.8, *p* < 0.001), while those in the Professional sector were 1.0 years older (*t* = 4.6, *p* < 0.001).

### 3.2 Characteristics of the sample

All ABS workplace sectors were represented with the largest numbers of respondents coming from the female dominated ‘Healthcare and Social Assistance’ sector (35.6%, *n* = 874) and the more equally gendered ‘Professional & Finance’ sector (27.2%, *n* = 669). All respondents were in paid employment with the majority in part‐time employment (64.2%, *n* = 1577). In total, just over half (56.5%, *n* = 1387) of all respondents had autonomy in planning their workday ‘most’ or ‘all of the time’, however this varied by workplace sector (see Table 2) with those in more male dominated industries having more autonomy.

**Table 2:** Characteristics across workplace sectors.

	Healthcare & Social Assistance		Education & Training		Administrative & Support Services		Professional & Financial		Mining & Manufacturing		Full sample	
Characteristic	*n*	%	*n*	%	*n*	%	*n*	%	*n*	%	*n*	%
**Coworker gender (2334)**												
Mostly female	629	76.1	302	75.5	159	49.7	246	38.4	27	18.5	1363	58.4
Equal male and female	179	21.6	92	23.0	113	35.3	276	43.1	43	29.5	704	30.2
Mostly male	19	2.3	6	1.5	48	15.0	118	18.4	76	52.0	267	11.4
**Manger gender (2440)**												
Mostly female	556	64.0	237	56.0	121	36.1	186	28.1	12	8.1	1112	45.6
Equal male and female	179	20.6	108	25.5	105	31.3	207	31.2	30	20.8	630	25.8
Mostly male	134	15.4	78	18.4	109	32.5	270	40.7	107	71.8	698	28.6
**Worker autonomy (2442)**												
Yes	387	44.4	171	40.5	234	70.3	481	72.4	114	75.5	1387	56.8
No	485	55.6	251	59.5	99	29.7	183	27.6	37	24.5	1055	43.2
**Confident in maintaining supply (*n* = 1818)**												
Yes	494	71.4	180	66.4	166	70.0	377	75.0	91	77.8	1309	72.0
No	198	28.6	91	33.6	71	30.0	123	25.0	26	22.2	509	28.0
**Formal RTW plan (*n* = 2452)**												
Yes	366	42.0	191	45.0	181	53.9	314	47.1	82	54.0	1134	46.3
No	244	27.5	104	24.5	84	25.0	235	35.2	50	32.9	718	29.3
Unsure	262	30.0	129	30.4	71	21.1	118	17.7	20	13.2	600	24.5
**Met goals (*n* = 2343)**												
Yes	627	74.7	303	75.0	242	75.4	459	72.7	107	72.8	1739	74.2
No	75	8.9	44	10.9	23	7.2	64	10.1	10	6.8	216	9.2
No goals	137	16.3	57	14.1	56	17.5	108	17.1	30	20.4	388	16.6

Abbreviation: RTW, Return To Work.

All participants (*n* = 2457) had RTW and breastfed in the last 3 years. Slightly less than half of the participants (44%, *n* = 1087) were still breastfeeding at the time of the survey, the rest had ceased within the last 3 years. More than 9 out of 10 women had support to breastfeed from the people they lived with (96.0%, *n* = 2359) and 91% (*n* = 2244) received support to breastfeed from their partner. The mean age of the child on RTW&BF was 8.6 months (*SD* 3.7, range: 1 week to 32 months). Of those who had ceased breastfeeding, the mean length of time breastfeeding their youngest child was 19.3 months (*SD* 8.8, range: 2 weeks to 6 years).

To maintain breastmilk supply, almost three quarters of women EBM at work (70.7%, *n* = 1737). More than half of participants combined breastfeeding and expressing of breastmilk (56.7%, *n* = 1392) after RTW and a small number did not need to express breastmilk for their infant (21.1%, *n* = 519). Of the women who had ceased EBM (*n* = 1234), the mean length of time EBM at work was 5.8 months (*SD* 4.6, range: 0.1–48 months) and the mean age of infants when women no longer needed to express milk at work was 13.5 months (*SD* 4.3, range: 1–36 months).

When at work, two thirds of women identified a childcare centre as caring for their child (67.8%, *n* = 1665). Only 12.1% (*n* = 299) of respondents had a workplace childcare centre available. Three‐quarters of respondents had previously RTW while maintaining breastfeeding for another child (72.2%, *n* = 1773), but almost one‐third of respondents (27.8%, *n* = 684) were navigating this experience with their first child.

The majority of women indicated that they worked in employment sectors comprised of mostly female co‐workers or equally male/female co‐workers (Table 2). Just under half of respondents (45.3%, *n* = 1112) reported having a female manager and similar proportions had either male managers (28.4%, *n* = 698) or an equal proportion of male or female managers (25.6%, *n* = 630). Less than one‐third (29.2%, *n* = 718) of respondents reported having a formal RTW plan in their workplace, with one quarter (24.4%, *n* = 600) indicating that they were ‘unsure’ about this. When those with a RTW plan were asked whether breastfeeding was specified in the RTW plan, 22.6% (*n* = 298) selected ‘no’ and half were ‘unsure’ (48.3%, *n* = 636).

The data were analysed to determine factors influencing a women's level of confidence in: maintaining breastmilk supply after RTW; whether they met their own breastfeeding goals; and whether there were differences in confidence maintaining breastmilk supply or meeting breastfeeding goals across the different work sectors.

### 3.3 Factors influencing confidence to maintain breastmilk supply after RTW

In total, 1818 mothers responded to the questions relating to breastfeeding confidence, of these almost three quarters (72.0%) reported they were confident (‘very confident’ or ‘somewhat confident’) in maintaining their breastmilk supply after RTW.

Participants who reported confidence in maintaining breastmilk supply were more likely to report a high level of confidence in speaking up about breastfeeding rights at work (MW‐U std score = 10.02, *p* < 0.0001). Other factors linked to confidence in maintaining breastmilk supply included: RTW when their infant was older, breastfeeding overall for a longer period, and a higher level of support for breastfeeding at home (see Table 4). Working fulltime, part time or casual did not impact confidence in maintaining milk supply.

Forty‐one percent (of *n* = 1825) reported that they did not have a suitable place to express breastmilk, and 7% did not have access to a fridge. In response to an open text ‘other’ option, some participants (17% *n* = 317) reported using spaces such as their car, a toilet cubicle, a storage cupboard or the sick bay (first aid room) as a place to express milk. Most participants (72.0% *n* = 1621) reported that, at times, they were *unable* to take their scheduled lactation breaks due to work commitments. This was significantly related to worker autonomy: 52% of workers *with* autonomy were ‘sometimes’ unable to take breaks, compared to 69% of workers without autonomy (Chi.Sq. = 50.5, *df* = 1, *p* < 0.001). Always being able to take breaks was related to being more confident in maintaining milk supply, with 84% of those always permitted to take lactation breaks being confident, compared to 69% of those sometimes limited in taking their breaks (Chi.Sq. = 29.8, *df* = 1, *p* < 0.001). Less than half of the participants (48% *n* = 1169) reported that their workplace recognised the importance of breastfeeding.

Of note, participants who were confident in maintaining milk supply were *more likely to have* managers who were equal male and female. The proportion of participants with confidence in maintaining milk supply was lowest for participants whose managers were mostly male (69%, compared to 76%; Table 3). Participants were also more confident in maintaining milk supply if they had a suitable place to EBM that met their needs (Chi.Sq. = 101.4, *df* = 1, *p* < 0.001) and had access to a fridge (Chi.Sq. = 5.95, *p* = 0.051). Not being able to take breaks was more detrimental to confidence in maintaining milk supply than access to a fridge (see Table 4).

**Table 3:** Scale variables by workplace sector.

		Healthcare & Social Assistance		Education & Training		Administrative & Support Services		Professional & Financial		Mining & Manufacturing		Full sample		ANOVA	
Characteristic	*n*	Mean	*SD*	Mean	*SD*	Mean	*SD*	Mean	*SD*	Mean	*SD*	Mean	*SD*	*F*	*p*
Maternal age	2451	33.0	4.1	32.8	4.2	31.4	4.5	34.0	4.4	33.5	4.6	33.0	4.3	21.7	<0.0001
Confidence in speaking up about rights	2146	75.5	23.0	68.2	26.3	74.6	25.3	77.2	22.7	76.9	23.8	74.7	24.1	9.12	<0.0001
Age ceased BF (months)^a^	1025	19.4	8.6	18.7	9.1	19.2	7.9	20.0	9.7	16.4	6.8	19.2	8.8	2.14	0.074
Age of child when RTW^a^	2440	8.8	3.8	8.8	3.8	7.9	3.6	8.8	3.7	7.8	3.5	8.6	3.7	5.82	0.0001
Length of time EBM at work (months)^a^	1234	5.7	4.6	5.3	3.9	6.6	5.2	5.8	4.8	5.4	3.3	5.8	4.5	1.82	0.122
Age of child when EBM no longer needed (months)^a^	1213	13.6	4.2	12.8	4.0	13.5	4.2	13.9	4.7	12.8	3.6	13.5	4.3	2.71	0.029

Abbreviations: ANOVA, analysis of variance; EBM, expressed breastmilk; RTW, Return To Work.

^a^ Results are strongly cross correlated, absolute *r* values range from 0.25 to 0.52.

**Table 4:** Factors influencing confidence in milk supply and meeting goals.

	Confidence in maintaining supply (*n* = 1818)					Met goals (*n* = 2343)				
Demographic and workplace factors	*n*	*n* conf	% conf	Chi.Sq.	*p*	*n*	*n* met	% met	Chi.Sq.	*p*
**Country of birth**										
Australia	1817	1138	72.0	0.00	0.987	2341	1516	74.9	2.80	0.09
Other		170	71.7				222	70.3		
**Geographical remoteness**										
Urban	1727	288	74.4	1.74	0.420	2237	360	73.6	0.18	0.91
Suburban		652	70.9				853	74.4		
Rural		304	72.4				442	73.5		
**Education**—**high school**										
Post‐school	1816	1195	71.7	0.72	0.397	2340	1592	74.3	0.02	0.90
School		113	75.3				145	73.6		
**Education—university**										
Yes	1818	951	72.3	0.18	0.668	2343	1250	74.6	0.43	0.51
No		358	71.2				489	73.2		
**Work sector**										
Health	1817	494	71.4	9.57	0.048^a^	2342	627	74.7	1.35	0.85
Educ.		180	66.4				303	75.0		
Admin.		166	70.0				242	75.4		
Prof.		377	75.4				459	72.7		
Mining		91	77.8				107	72.8		
**Coworker gender**										
Mostly female	1740	699	70.6	2.28	0.32	2230	969	74.8	1.61	0.45
Mixed		394	73.0				490	72.4		
Mostly male		158	75.2				194	75.5		
**Manager gender**										
Mostly female	1812	575	71.3	6.46	0.040^a^	2330	782	74.1	0.07	0.97
Mixed		349	76.4				449	74.5		
Mostly male		380	69.3				496	73.8		
**Return to work plan**										
Yes	1818	407	76.5	10.89	0.004^a^	2340	519	76.0	11.7	0.003
Unsure		319	73.3				444	78.2		
No		583	68.5				773	71.0		
**Worker autonomy**										
Yes	1809	785	77.3	30.53	<0.001^a^	2333	1001	75.8	3.58	0.059
No		520	65.5				731	72.2		
**Unable to take breaks at times due to work**										
Yes	1764	325	83.6	29.8	<0.001^a^	1762	495	78.7	9.2	0.002
No		954	69.4				1171	72.3		
**BF support at home**										
Yes	1818	1273	72.5	4.63	0.031^a^	2343	1675	74.4	1.2	0.27
No		36	59.0				64	68.8		
**A suitable place to EBM**										
Yes	1818	863	81.0	101.4	<0.001^a^	1784	822	78.1	27.6	<0.0001
No		446	59.3				489	66.8		
**Access to a fridge**										
Yes	1812	1205	72.9	5.95	0.051	1780	1206	74.1	3.02	0.221
Unsure		19	76.0				16	72.7		
No		84	63.2				88	67.2		
**Workplace support RTWBF**										
Yes	1811	703	78.3	42.1	<0.001^a^	2334	889	79.0	29.6	<0.0001
Unsure		275	70.5				390	72.4		
No		325	62.4				452	67.6		

		Conf.	Not conf.				Met goals	Did not met goals/no goals		
	*n*	Mean (*SD*)	Mean (*SD*)	Std MW‐U	*p*	*n*	Mean (*SD*)	Mean (*SD*)	Std MW‐U	***p***
Maternal age (years)	1815	33.1 (4.3)	32.5 (4.3)	2.48^b^	0.014	1812	33.1 (4.3)	32.8 (4.5)	−1.78^b^	0.075
Confident to speak about rights	1663	79.3 (20.7)	65.9 (26.4)	10.02	<0.001	1617	77.2 (22.4)	67.4 (27.2)	7.46	<0.001
Age of child at return to work^c^	1809	8.4 (3.5)	7.7 (3.2)	3.96	<0.001	1803	8.7 (3.7)	8.2 (3.6)	3.33	<0.001
Length of time EBM at work^c^	1223	6.2 (4.8)	4.7 (3.8)	6.43	<0.001	681	6.2 (4.8)	4.7 (3.7)	6.07	<0.001
Child age when EBM no longer needed^c^	1212	13.9 (4.3)	12.4 (4.0)	6.03	<0.001	667	14.1 (4.2)	12.4 (4.3)	7.58	<0.001
Age of youngest child when ceased BF^c^	764	20.6 (9.3)	15.7 (6.9)	7.44	<0.001	446	20.5 (8.9)	16.1 (8.0)	7.98	<0.001

*Note*: % = Percentage.

Abbreviations: BF, breastfeeding; Chi.Sq., Chi Squared test statistic; conf, confident in maintaining breastmilk supply; EBM, expressing breastmilk; met, mother met breastfeeding goals; *n*, number of participants; *SD*, standard deviation; Std MW‐U, standardised test statistic associated with Mann–Whiney *U* test.

a Significant at the 0.05 level.

^b^ *t*‐test performed and reported as data normally distributed.

^c^ Factors moderately to strongly cross‐correlated (rho = 0.31–0.63).

### 3.4 Factors influencing breastfeeding goal attainment

A total of *n* = 2343 participants responded to the question on breastfeeding goals. Most reported that they were able to meet their own breastfeeding goals (74.2%, *n* = 1739), although 16.6% (*n* = 388) of participants indicated that they did not have any breastfeeding goals.

Women were more likely to meet their breastfeeding goals when they had a formal RTW plan in the workplace (Chi.Sq. = 27.6, *p* < 0.001) and were able to take breaks when they needed to (Chi.Sq. = 9.2, *p* = 0.002). Having a suitable place to express milk (Chi.Sq. = 11.7, *p* = 0.003), a workplace that supports RTWBF (Chi.Sq. = 29.6, *p* < 0.001), and having confidence in standing up for rights (Std MW‐U. = 7.46, *p* < 0.001) were key predictors for meeting breastfeeding goals. Overall, participants who had met their breastfeeding goals RTW when their infant was slightly older (mean: 8.7 months compared to 8.2 months; *p* < 0.001) and breastfed their infant for longer (mean: 20.5 months compared to 16.1 months, *p* < 0.001), compared to participants who felt that they did not meet their breastfeeding goals (Table 4).

### 3.5 Work sector differences

Participants in the female dominated ‘Education and Training’ sector and the ‘Health care and Social Assistance’ sectors reported less autonomy than those in other workplace sectors (see Table 2). Those employed in the ‘Education and Training’ sector also had less confidence in speaking up about their rights to breastfeed at work (mean score: 68.2 out of a possible score of 100, *F* = 9.12, *p* < 0.001). Overall women in the ‘Administrative and support services’ sector and the ‘Mining and manufacturing’ sector groupings RTW earlier than those in the other workplace sectors. Confidence in maintaining breastmilk supply was significantly lower in the female dominated ‘Education and training’ group compared to women in the ‘Mining and manufacturing’ and ‘Professional’ workplace groupings. Despite these differences there were no significant differences in breast‐feeding duration, length of time expressing breastmilk at work, or meeting personal breastfeeding goals across all workplace sectors (see Table 4).

### 3.6 Regression modelling: Key predictors for confidence in supply and meeting goals

#### 3.6.1 Confidence in maintaining breastmilk supply

##### Regression model 1a

Backward logistic regression found five key predictors of being confident in maintaining breastmilk supply (Table 5), these included being able to take breaks when needed, having a suitable place to EBM, confidence in breastfeeding rights, and age of child at RTW and length of time EBM at work. Women who could take their lactation breaks when they needed had 1.8 times the odds of being confident in maintaining supply than those who could not. Similarly, the odds of a woman who had a suitable place to EBM were 2.4 times higher, for confidence in maintaining breastmilk supply, compared to those with no suitable place to EBM. Each 1‐point rise in confidence in speaking out about breastfeeding rights was associated with a 2% increase in level of confidence in maintaining supply.

**Table 5:** Logistic regression showing key factors affecting confidence in maintaining milk supply and breastfeeding goal achievement.

		Confidence in maintaining supply (*n* = 1818)						Met breastfeeding goals (*n* = 2343)					
		model 1a: *n* = 1032			model 1b: *n* = 1630			model 2a: *n* = 1038			model 2b: *n* = 1638		
Selected factors	Categories	inc	*p*	OR (95% CI)	inc	*p*	OR (95% CI)	inc	*p*	OR (95% CI)	inc	*p*	OR (95% CI)
Work sector	a	y	–	–	y	–	–	n			n		
Manager gender	^a^	y	–	–	y	–	–	n			n		
Return to work plan	Yes	y	–	–	y	–	–	y	–	–	y	0.695	0.95 (0.72–1.25)
	Unsure	y	–	–	y	–	–	y	–	–	y	0.039	1.37 (1.02–1.84)
Worker Autonomy	Yes	y	0.156	1.25 (0.92–1.69)	y	0.012	1.36 (1.07–1.72)	y	–	–	y	–	–
Take breaks	Yes	y	0.006	1.76 (1.18–2.67)	y	0.002	1.66 (1.22–2.28)	y	–	–	y	–	–
BF support at home	Yes	y	–	–	y	–	–	n			n		
A suitable place to BF	Yes	y	<0.001	2.36 (1.75–3.20)	y	<0.001	2.24 (1.77–2.84)	y	<0.001	1.67 (1.25–2.24)	y	0.003	1.44 (1.13–1.82)
Access to a fridge	Yes	y	–	–	y	–	–	n			n		
Workplace support RTWBF	Yes	y	–	–	y	–	–	y	–	–	y	0.048	1.33 (1.00–1.75)
	Unsure	y	–	–	n			y	–	–	y	0.654	1.08 (0.78–1.48)
Maternal age		y	–	–	y	–	–	y	–	–	y	0.101	1.02 (1.00–1.05)
Confident to speak about rights		y	<0.001	1.02 (1.01–1.03)	y	<0.001	1.02 (1.01–1.02)	y	<0.001	1.01 (1.00–1.02)	y	<0.001	1.01 (1.01–1.02)
Age of child at return to work		y	<0.001	1.14 (1.08–1.20)	y	<0.001	1.07 (1.04–1.11)	y	–	–	y	0.048	1.04 (1.00–1.07)
Length of time EBM at work		y	<0.001	1.11 (1.06–1.16)	n			y	0.155	1.03 (0.99–1.07)	n		
Child age when EBM no longer needed		y	–	–	n			y	<0.001	1.11 (1.06–1.16)	n		

*Note*: All models use backward step‐wise regression, factors not significantly contributing to the final fit are removed and shown with a hyphen. Models 1a and 2a had all variables shown as significant in Table 3 (except for ‘Age of youngest child when ceased BF’ due to a small sample size). Models 1b and 2b had two additional variables removed due to small sample size and potential confounding of the causal pathway between exposures and outcomes. All variables removed were strongly correlated with ‘Age of child at return to work’. OR = odds ratio, calculated by taking the exponent of β.

Abbreviations: CI, confidence interval; BF, breastfeed; EBM, express breastmilk; inc, variable included within model; OR, odds ratio; RTW, Return to work.

^a^ Categories for these factors include all those listed in Table 3.

##### Regression model 1b

This model included two fewer input variables and consequently had a larger sample size (*n* = 1630). The model demonstrated the same variables as model 1a (see Table 5), as having the greatest impact on confidence in maintaining breastmilk supply, with the influence of worker autonomy gaining in significance. Workers with autonomy at work were 34% more likely to be confident in maintaining their breastmilk supply on RTW than those without autonomy at work.

#### 3.6.2 Met breastfeeding goals

##### Regression model 2a

Backward logistic regression found four key predictors of women meeting breastfeeding goals (Table 5), these included having a suitable place to EBM, confidence in breastfeeding rights, age of child when EBM was no longer needed and length of time EBM at work. Those who had a suitable place to EBM had 1.7 times the odds of meeting their breastfeeding goals compared to those who did not have a suitable place. Confidence in speaking out about breastfeeding rights was associated with a 1% increase in the chance of meeting their goals for every point of confidence. The age of the child when mothers no longer needed to express milk at work revealed that for every additional month of EBM, women were 11% more likely to meet their goals.

##### Regression model 2b.

As with the second regression modelling for confidence with supply, regression model 2b included two fewer input variables so had a larger sample size (*n* = 1638). This model found two of the same key predictor variables as for model 2a (see Table 5). The main key predictor for achieving breastfeeding goals was the woman's confidence in speaking about her rights, which was associated with a 1% increase in the odds of meeting her goals for every increase point of confidence. The second biggest predictor for achieving goals was having a suitable place to EBM. Women with a suitable place had 1.4 times the odds of meeting their breastfeeding goals. Model 2b revealed four new predictors for achieving goals: having a formal RTW plan, having a supportive workplace, maternal age and age of child when mother RTW (see Table 5).

## 4 DISCUSSION

This is the largest known study of women's experiences of RTW&BF across all work sectors conducted in Australia to date. This paper identifies the key factors impacting women's confidence in maintaining breastmilk supply, as well as factors impacting the achievement of personal breastfeeding goals after RTW. These go beyond the traditional provision of access to a breastfeeding space, and time to express breastmilk, to include confidence in standing up for breastfeeding rights, autonomy, or flexibility at work for lactation breaks, and RTW when the infant is older (mean age: 8.4 months). Importantly, modifiable factors identified as impacting confidence and goal attainment include having a formal RTW plan, working in a supportive environment, and having support for breastfeeding in the home. Across Australian work sectors, women in the female dominated professions had less autonomy in planning their workday. Those in education and training were less confident in maintaining breastmilk supply compared to other sectors, whereas those working in the male dominated mining and manufacturing sector RTW earlier and ceased breastfeeding sooner than other sectors. Of note, women who worked in environments with a larger proportion of equal male and female employees had more confidence in maintaining milk supply. In addition, women who met their breastfeeding goals were more likely to breastfeed for longer than those who had not met their goals.

The key modifiable influences on breastfeeding confidence and achieving goals can be categorised as public policy, workplace and individual factors. These modifiable influencers include: (1) Availability of national paid parental leave; (2) Workplace factors such as having a space to express or feed, having flexibility for lactation breaks and a supportive work environment; and (3) Individual modifiable factors, within a woman's control, such as her knowledge of, and level of confidence with, breastfeeding rights in the workplace.

### 4.1 National factors: Paid maternity leave

Access to adequate paid maternity leave enables more women to meet the WHO recommendations of exclusive breastfeeding to 6 months and continued breastfeeding into the infant's second year (Chai et al., 2018; Lauzon‐Guillain et al., 2019). Given that Australian national paid maternity leave currently provides a maximum of 18 weeks leave, many women will RTW before their infant is 6 months old. Access to maternity leave is a proven determinant for increasing the duration and exclusivity of breastfeeding (Smith, McIntyre, et al., 2013; Stewart, 2015).

Australia currently provides paid maternity leave for 18 weeks, which is beyond the International Labour Organisation (ILO) accepted minimum of 14 weeks (ILO, 2000). However, maternity leave that covers the period of exclusive breastfeeding is predictive of longer breastfeeding durations (Steurer, 2017). Ahmadi and Moosavi (2013) found that women who had maternity leave of less than 6 months had higher use of infant formula than mothers with maternity leave greater than 6 months. Chai et al. (2018) compared data from 38 low‐ and middle‐income countries and found that every 1‐month increase in legislated maternity leave created a 5.9% increase in exclusive breastfeeding and a 2.2‐month increase in the overall duration of breastfeeding. Similar results have been reported in high income countries. After the introduction of paid maternity leave in California United States, exclusive breastfeeding rates increased by 3%–5% and the duration of breastfeeding increased by 10%–20% (Huang & Yang, 2015).

We welcome the proposed Australian legislation to change the paid parental leave to 20 weeks with an increase of 2 weeks each year until 26 weeks in 2026 (Services Australia, 2022). However, we call on the Australian government to ratify the ILO Maternity Protection Convention 183 and enshrine women's rights to paid maternity leave and paid workplace lactation breaks in legislation (ILO, 2000). While these are recommended practices for best practice employers in Australia (Fair Work Ombudsman, 2022), we argue that all mothers should have access to paid lactation breaks regardless of their employment status or employer.

### 4.2 Workplace factors: Having a suitable space to express milk or breastfeed

Access to a suitable place to express breastmilk is crucial to confidence in maintaining milk supply and meeting breastfeeding goals. Pumping breastmilk has been reported by women as “difficult”, “time consuming” and “unpleasant” compared to feeding at the breast (Felice et al., 2017; Henry‐Moss et al., 2018) therefore, providing a space where women feel comfortable in the workplace is vital for maintaining breastfeeding. The provision of space for breastfeeding communicates implicit recognition of the importance of breastfeeding (Dinour & Szaro, 2017) and presents breastfeeding as a normal part of working life for childbearing women.

A recent systematic review of interventions for RTW&BF revealed that the most common intervention across 37 studies was provision of a space for breastmilk expression (Vilar‐Compte et al., 2021). Simply having knowledge of a dedicated workspace to breastfeed, or express milk, can lead to higher odds ratios of continuing breastfeeding after RTW (Chen et al., 2006). Evidence reveals that women without access to a lactation space, and a fridge to store expressed milk, can be 1.8 times more likely to stop breastfeeding after RTW (Dinour & Szaro, 2017).

Proximity of access to a breastfeeding space is also crucial. Gilmour et al. (2013) explored RTW&BF experiences at an Australian university and found that provision of a lactation room was inadequate if it was located too far from where the woman worked. The importance of proximity was also reported in research by Henry‐Moss et al. (2018) where participants indicated the maximum walking distance to the lactation space should be no more than 5.6 min. Although our study did not investigate proximity, we can report that simply providing a space that was comfortable and met the woman's needs, including access to a fridge to store milk, led to increased confidence in maintaining supply and longer duration of expressing breastmilk at work.

### 4.3 Workplace factors: Having autonomy or flexibility in the workday

Flexible work options were highlighted as an important facilitator for maintaining milk supply. Being able to work part‐time, work from home and have access to maternity leave is known to improve the duration of breastfeeding (Smith, Javanparast, et al., 2017; Stewart, 2015). Australian research by Xiang et al. (2016) reveals that older maternal age, higher educational attainment, more senior occupational status, or being self‐employed, led to increased likelihood of RTW and breastfeeding. Women in professional or managerial positions tend to have access to the most support on RTW when compared to women in the service industries (Snyder et al., 2018). Research in a Spanish university (Leon‐Larios et al., 2019) revealed the ease with which academic staff could arrange to take expressing breaks, compared to administrative staff, which resulted in higher continuation of breastfeeding for academic staff. This resonates with the findings from our study where women with flexibility in planning their workday had more confidence and met their goals.

Autonomy at work is associated with plans to RTW&BF. Women with higher autonomy during pregnancy are more likely to set an intention to RTW and maintain breastfeeding (Spitzmueller et al., 2018). This reinforces the importance of supporting those in low autonomy positions to have access to workplace provisions. Health professional staff, who promote exclusive breastfeeding to women, report that they often could not maintain exclusivity after RTW themselves due to the nature of their work and inability to take scheduled breaks (Gebrekidan et al., 2021). Women in customer‐facing low autonomy roles are often denied access to a staff member to cover their break (Dixit et al., 2015). Exploration of breastfeeding support for trainee paediatric doctors in the United States revealed that they experienced co‐worker “resentment’ for picking up the “slack”, when expressing breastmilk, and this made the breastfeeding woman feel inadequate and unable to maintain supply. Many of the breastfeeding trainee doctors concluded that RTW&BF was not compatible with long shifts and lack of cover for breaks. Unmet breastfeeding goals led to negative feelings about RTW&BF, with these clinicians becoming less inclined towards promoting breastfeeding (Dixit et al., 2015). A workplace that undermines a woman's confidence in maintaining milk supply can lead to low job satisfaction and work‐family conflict, potentially impacting psychosocial wellbeing. Dixit et al. (2015) report that for trainee doctors who had a positive RTW experience, and support to meet breastfeeding goals, this led to greater promotion of breastfeeding.

### 4.4 Workplace factors: Having a supportive workplace with a formal RTW policy

Evidence demonstrates that supportive breastfeeding friendly workplaces are rewarded with fewer sick days for breastfeeding workers, with less need to take personal leave to care for a sick baby, as breastfeeding provides protective factors for both maternal and infant health (Smith, McIntyre, et al., 2013). Workplace culture requirements include the importance of policy provisions and supportive co‐workers and managers. Having peers and managers who are knowledgeable about breastfeeding rights and who proactively support breastfeeding workers reflects an accepting workplace culture. Open communication between managers and workers can facilitate a smooth transition to RTW&BF (Gilmour et al., 2013).

Workplace impediments, to ongoing breastfeeding, permeate all organisations. Even breastfeeding workers in the WHO Western Pacific offices have reported barriers to RTW and breastfeeding (Iellamo et al., 2015). Having policies is one thing but ensuring they are enacted is another. Policewomen report that having a policy is important but only if is accompanied with a supportive work culture (Newton & Huppatz, 2020). Without support and promotion, the policy is merely a ‘cosmetic’ box ticking exercise. Even when policies are in place, there is often a policy—practice gap where the policy disregards the realities of maintaining breastfeeding in the workplace.

Whilst work colleagues can offer support to breastfeeding women, managers are often focused on outputs and this can impact the level of support provided (Bai, Wunderlich, et al., 2012; Gebrekidan et al., 2021). Research by Zhuang et al. (2018) in the United States reported that whilst the majority of co‐workers are likely to be supportive of RTW&BF, at least one in four workers stigmatised the breastfeeding women and resented the additional lactation breaks. Pregnant women assess workplace support for breastfeeding, during pregnancy, and this impacts the setting of breastfeeding goal intentions. Negative comments about breastfeeding from work colleagues and managers can lead to earlier cessation of breastfeeding (Spitzmueller et al., 2016).

Meeting the needs of breastfeeding workers and creating a supportive culture can be driven by workplace accreditation schemes. In Australia, Breastfeeding Friendly Workplace (BFW) Accreditation is managed by the Australian Breastfeeding Association (ABA, 2022b). Currently more than 130 workplaces have achieved BFW Accreditation (ABA, 2022a). In one university, which previously had ABA BFW Accreditation, the lack of re‐accreditation led to a decrease in organisational support for breastfeeding women and unmet personal breastfeeding goals (Smith, Javanparast, et al., 2017). Receiving, and renewing, BFW accreditation ensures that provisions for breastfeeding workers are available more than on an ‘ad hoc’ basis.

Our research concurs with Vilar‐Compte and colleagues (2021) that confidence in maintaining milk supply after RTW is dependent on several factors including: type of workplace and level of autonomy, support from partner and family, support from co‐workers and managers and individual assertiveness. Yet responsibility for enabling breastfeeding after RTW does not rest with workplaces alone. Health professionals have extended contact with women during pregnancy and after birth. This is an opportune time to educate women about their breastfeeding rights and to encourage those planning to RTW to consider options for maintaining breastmilk supply.

### 4.5 Social factors: Developing confidence in standing up for breastfeeding rights

Our study included a large proportion of university educated women who reported a belief in their rights to breastfeed for as long as they desired. This university educated, and confident, cohort of women were more likely to have positive support from their partner, and family, and breastfed for longer durations than women with lower levels of confidence in maintaining milk supply. Women who reported low confidence in maintaining milk supply after RTW (48%) tended to be less confident about asserting their rights at work and were less likely to have a partner who supported breastfeeding, possibly leading to breastfeeding cessation earlier than those who had confidence in maintaining supply. This study demonstrates that improving women's confidence in their breastfeeding rights can ultimately impact confidence in maintaining breastmilk supply, and meeting personal breastfeeding goals, after RTW.

Feeling confident in achieving personal breastfeeding goals is directly linked to extended breastfeeding duration (Sriraman & Kellams, 2016). While workplace support can have a mediating effect on this, intention to RTW&BF can positively impact breastfeeding duration (Wallenborn et al., 2019). Not all women will RTW in the infants' first year, yet it is important that conversations about RTW&BF become an integral part of health professional conversations during pregnancy and in the early post birth period, as appropriate.

If RTW&BF is challenging for the well‐educated assertive Australian‐born women in this study, then the experiences of others who do not fit this description may be much worse. Research by Brown (2014) revealed that personality traits can also impact breastfeeding confidence. Women who are introverted or have high levels of anxiety are less likely to stand up for their rights to breastfeed and tend to have lower breastfeeding duration. Our study identified additional groups that were less confident in maintaining milk supply for their infant. These included women with unsupportive partners or lack of family support, and women in low autonomy workplace positions.

It is illegal in Australia to discriminate on the grounds of breastfeeding, however our study has revealed that women still need to stand up for this fundamental human right. This study highlights the many groups of women who were unable to meet their personal breastfeeding goals due to workplace barriers. We agree with Brown (2018) that in order “…to move forward we must invest in mothers” with targeted social support options for those who are at highest risk of not meeting breastfeeding goals. Creating supportive social networks, online, at workplaces, and within the community, may enable individual women to build confidence in standing up for their rights to breastfeed or express milk after RTW.

### 4.6 Limitations

This study was an online survey so it was only accessible to those who could speak English and had sufficient literacy. Representation of migrant and refugee women, non‐English speaking mothers, workers in low autonomy roles and those in less secure casualised positions were underrepresented in this study. Participants were recruited after they had RTW within the last 3 years and, therefore, there is a risk of recall bias in reporting their experience. The survey was developed as part of an exploratory study and there are currently no Australian validated tools to measure the concepts we were exploring. The survey was promoted through the Australian Breastfeeding Association social media platforms which may have resulted in the over representation of a committed and assertive breastfeeding cohort.

## 5 CONCLUSION

Bolstering a woman's confidence in maintaining breastmilk supply to meet her breastfeeding goals is important for the achievement of WHO targets for 50% exclusive breastfeeding by 2025. Building women's confidence in her right to continue breastfeeding after RTW and improving workplace factors: such as flexible work arrangements; suitable place to express breastmilk; and a supportive work culture, can help facilitate continued breastfeeding after RTW. National and workplace investment in supporting women to maintain breastfeeding after RTW will have positive implications for the health and wellbeing of the breastfeeding woman, her infant, family, and community.

## AUTHOR CONTRIBUTIONS

Elaine Burns, Heather Pierce, Sky Hugman, Susanne Gannon conceptualised and designed the study and survey tool. Elaine Burns, Emma Elcombe and Heather Pierce analysed the data. Elaine Burns and Emma Elcombe wrote the paper. Heather Pierce, Sky Hugman and Susanne Gannon reviewed and edited several drafts of the manuscript. All authors reviewed and accepted the final version of the manuscript submitted to the journal.

## CONFLICT OF INTEREST STATEMENT

The authors declare no conflicts of interest.

## Data Availability

Data available on request from the authors.
